# Association between acrylamide exposure and sex hormones in males: NHANES, 2003–2004

**DOI:** 10.1371/journal.pone.0234622

**Published:** 2020-06-18

**Authors:** Pei-Lun Chu, Hui-Shan Liu, Chikang Wang, Chien-Yu Lin

**Affiliations:** 1 School of Medicine, College of Medicine, Fu Jen Catholic University, New Taipei, Taiwan; 2 Department of Internal Medicine, Fu Jen Catholic University Hospital, Fu Jen Catholic University, New Taipei, Taiwan; 3 Department of Gynecology and Obstetrics, Hsinchu Cathay General Hospital, Hsinchu, Taiwan; 4 Department of Environmental Engineering and Health, Yuanpei University of Medical Technology, Hsinchu, Taiwan; 5 Department of Internal Medicine, En Chu Kong Hospital, New Taipei, Taiwan; University Hospital of Münster, GERMANY

## Abstract

**Introduction:**

Acrylamide is widely present in heat-processed food, cigarette smoke and environment. Reproductive toxicity was reported in animals treated with acrylamide, particularly in males. The reproductive toxicity of acrylamide and its active metabolite, glycidamide, was reported to be mainly mediated through DNA damage in spermatocytes. However, the effect of acrylamide on sex hormones in men is unknown.

**Methods:**

There were 468 male subjects (age ≧ 12 years) enrolled to determine the relationships between hemoglobin adducts of acrylamide (HbAA) and hemoglobin adducts of glycidamide (HbGA) with several sex hormones using the National Health and Nutrition Examination Survey (NHANES), 2003 to 2004. All potential confounding variables in the data set were properly adjusted.

**Results:**

We found that one unit increase in the natural log-transformed HbAA level was associated with an increase in natural log transformed serum inhibin B level by 0.10 (SE = 0.05; *P* = 0.046), and natural log transformed serum sex hormone binding globulin (SHBG) by 0.15 (SE = 0.15; *P* = 0.036). With respect to HbGA, one unit increase in the natural log-transformed HbGA level was associated with an increase in natural log transformed serum anti-Müllerian Hormone (AMH) level by 0.31 (SE = 0.00; *P* = 0.003).

**Conclusion:**

In this representative cohort, we identified positive associations between acrylamide exposure and several sex hormones in men. The HbAA is positively associated with inhibin B and SHBG, and HbGA is positively associated with AMH. Other than genotoxicity, our findings suggested that altered sex hormones might also play a role in acrylamide-related reproductive toxicity in males.

## Introduction

Acrylamide is a chemical reactive monomer that is widely present in industries. It is also found in carbohydrate-rich foods processed at high temperatures and cigarette smoke.[1] In animals and humans, acrylamide is metabolized to glycidamide, which is subsequently hydrolyzed and conjugated with glutathione.[1] With chronic exposure, the hemoglobin adduct derived from acrylamide metabolism, acrylamide (HbAA) and glycidamide (HbGA), can be used as surrogates of acrylamide exposure in epidemiological studies.[2]

Acrylamide causes tumors in animas and has been classified as a probable human carcinogen.[1] Intriguingly, many of these tumors, such as thyroid tumors, pheochromocytomas and mammary gland tumors, are hormone sensitive. Additionally, acrylamide was also reported to be associated with neurotoxicity and reproductive toxicity.[3] Acrylamide exposure could lead to reduced fertility, embryo implantation defects and shorter postnatal survival in animals.[4] When acrylamide is metabolized, the acrylamide-to-glycidamide conversion could impair sister chromatid exchange and lead to glycidamide-DNA adducts formation and causing subsequent DNA damage that predominantly affects male animals.[5] In women, it has been shown that dietary acrylamide intake was associated with sex hormone alterations.[6] These results suggest sex hormone alteration could be an alternative mechanism of acrylamide’s reproductive toxicity other than genotoxicity.

Collectively, acrylamide exhibited genotoxicity predominately in male animals and is association with sex hormone alternations observed mainly in females. The association between acrylamide and sex hormone alternations in human males remains unclear. Herein, we utilized the 2003–2004 National Health and Nutrition Examination Survey (NHANES) to explore the association between acrylamide exposure and sex hormones in men to further understand the potential role of acrylamide in reproductive toxicity in human.

## Materials and methods

### Study design and population

Data were acquired from the 2003–2004 NHANES, which is a population-based survey in U.S. households to acquire a representative sample of the noninstitutionalized U.S. civilians.[7] The analyses were limited to male participants older than 12 years with data including HbAA, HbGA, sex hormones and complete data on demographics, smoking status as well as BMI. This project was approved by the ethics committee and Institutional Review Board of the En Chu Kong Hospital (Reg. No. ECKIRB1080902), New Taipei City, Taiwan.

### Assessment of HbAA and HbGA

Briefly, the HbAA and HbGA levels were determined in 350 μL of whole blood and analyzed using high-performance liquid chromatography/tandem mass spectrometry. The total hemoglobin level was determined as cyanmethemoglobin using a commercially available assay (Stanbio Laboratory, USA). The detection thresholds were 3 pmol/g Hb for HbAA and 4 pmol/g Hb for HbGA, respectively. If the concentrations were below the detection thresholds (1.8% of blood samples for HbGA), an imputed value was assigned by the NHANES and adapted in our analyses. More detailed information is available on the NHANES website (https://www.cdc.gov/nchs/nhanes/index.htm).

### Assessment of sex hormones

The concentrations of testosterone, sex hormone binding globulin (SHBG), androstanediol glucuronide (a metabolite of dihydrotestosterone) and estradiol were measured in stored serum specimens. The serum testosterone, androstanediol glucuronide, SHBG and Estradiol II levels were measured using a commercial kit (Roche Diagnostics, Switzerland) based on electrochemiluminescence immunoassay. The levels of free and bioavailable testosterone were represented by the sum of the free and albumin-bound testosterone fractions, respectively.[8] The serum anti-Müllerian hormone (AMH) and inhibin B levels were measured using an enzyme-linked immunosorbent assay to evaluate variations mediated by age and race/ethnicity.

### Covariates

Sociodemographic information, including age, gender, and race/ethnicity, was recorded during the household interview. For participants older than 20 years, the BMI z-score was derived from the following equation: (BMI of each participant—mean BMI)/(standard deviation of BMI). For participants aged 12–19 years, the BMI z-score was calculated based on the WHO anthropometric calculator.[9] Smoking status was categorized as active smoker, environmental tobacco smoke exposure, or non-exposure by smoking questionnaires, and serum cotinine levels were determined by isotope dilution high-performance liquid chromatography / atmospheric pressure chemical ionization tandem mass spectrometry.[10] Active smoker was defined as individuals with serum cotinine levels > 15 ng/mL or those who reported currently smoking every day or on some days. Participants with detectable serum cotinine levels < 15 ng/mL and without current smoking history were categorized as environmental tobacco smoke exposure. Participants with undetectable serum cotinine levels (< 0.015 ng/mL), without reported smoking at home or self-reported smoking were categorized as non-exposure.

A two-day detailed dietary intake information from each participant was used to estimate the types and amounts of foods/beverages consumed during the 24-hour period prior to the interview. The first-day data were collected at the Mobile Examination Center, whereas the second-day data were collected via telephone later. The caffeine intake from the two days was averaged as a covariate in this study.

### Statistics

HbAA and HbGA concentrations were expressed as the geometric mean with a 95% confidence interval (CI) in different subgroups and tested by a 2-tailed Student’s t-test as well as one-way analysis of variance (ANOVA). Because of the significant deviation from normal distribution, the natural log-transformation of HbAA, HbGA, and sex hormones was employed. We used each sex hormone as a dependent variable and individual natural log-transformed HbAA and HbGA as predictors in the multiple regression analysis. The model was adjusted for age (continuous variable), race/ethnicity (categorical variable), BMI z-score (continuous variable), and smoking status (categorical variable). For further investigation of the associations between acrylamide exposure and sex hormones in subgroups, multiple linear regression analyses were performed. The analyses were carried out using sampling weights to examine the effects of weighting. Sampling weights were derived using procedures based on the National Center for Health Statistics analytic guidelines and properly accounted for the complex survey design of the NHANES 2003–2004.[11] Sampling weights accounting for unequal probabilities of selection, oversampling, and nonresponse were applied to all analyses using the complex sample survey module of SPSS Version 20 for Windows 7 (SPSS, U.S.A.). *P* < 0.05 was considered significant.

## Results

There were 468 male subjects older than 12 years in the study (S1 Fig). The basic demographics of the sample population and the association between acrylamide adducts are outlined in Table 1. HbAA and HbGA were detectable in 100% and 95.6% of the study subjects, respectively. The mean concentrations (95% C.I.) of HbAA and HbGA were 78.9 (72.82–85.01) pmol/g Hb and 69.7 (64.42–75.00) pmol/g Hb. HbAA and HbGA were strongly correlated (p<0.001), with a Spearman correlation coefficient of 0.81. The distribution of the sex hormones and the correlations between them were shown in S1 and S2 Tables. Participants between the ages of 20 and 45 years, lower BMI z-scores, active smoking habits, and higher caffeine intake were associated with higher HbAA and HbGA concentrations.

**Table 1:** Basic demographics of the sample subjects including means (95% C.I.) of acrylamide and glycidamide adduct concentrations.

	Unweighted no. (%)	HbAA (pmol/g Hb)	*P* value between groups	Unweighted no. (%)	HbGA (pmol/g Hb)	*P* value between groups
Overall	468 (100)	78.91 (72.82–85.01)		454 (100)	69.71 (64.42–75.00)	
Age, y			<0.001			<0.001
12–19	161 (34.4)	69.26 (59.17–79.35)		156 (34.4)	64.10 (55.29–72.91)	
20–44	133 (28.4)	100.69 (85.49–115.88)		130 (28.6)	86.48 (74.33–98.64)	
≧45	174 (37.2)	71.20 (62.67–79.73)		168 (37.0)	61.95 (54.14–69.75)	
Race			0.204			0.274
Mexican American	122 (26.1)	77.56 (65.65–89.47)		117 (25.8)	74.35 (63.93–84.76)	
Non-Hispanic White	201 (42.9)	77.95 (68.66–87.22)		198 (43.6)	70.03 (62.03–78.04)	
Non-Hispanic Black	113 (24.2)	87.88 (75.50–100.26)		108 (23.8)	68.49 (57.64–79.33)	
Others Hispanic	16 (3.4)	47.44 (14.54–80.35)		16 (3.5)	39.94 (11.81–68.08)	
Other race	16 (3.4)	69.62 (36.72–102.52)		15(3.3)	69.90 (40.84–98.96)	
BMI z score			<0.001			0.024
≦ 0.15	232 (50.1)	92.85 (84.33–101.37)		226 (50.3)	75.90 (64.41–83.40)	
>0.15	231 (49.9)	65.34 (56.80–73.88)		223 (49.7)	63.67 (56.12–71.21)	
Smoking			< 0.001			< 0.001
Nonexposed	64 (13.7)	50.28 (36.40–64.16)		60 (13.2)	52.37 (39.26–65.49)	
Exposed to ETS	264 (56.4)	56.39 (49.56–63.23)		257 (56.6)	53.49 (47.16–59.83)	
Active smoker	140 (29.9)	134.47 (125.09–143.86)		137 (30.2)	107.73 (99.05–116.41)	
Caffeine intake (mg/day)			0.006			0.004
< 72	205 (49.6)	68.27 (59.41–77.13)		199 (49.6)	61.08 (53.32–68.85)	
≧72	208 (50.3)	85.96 (77.04–94.89)		202 (50.4)	77.00 (69.30–84.71)	

The linear associations between levels of HbAA, HbGA and various sex hormones in sample subjects weighted for sampling strategy are shown in Table 2. For HbAA, one unit increase in the natural log-transformed HbAA level was associated with an increase in the natural log-transformed serum inhibin B level by 0.10 (SE = 0.05; *P* = 0.046) and the natural log-transformed serum SHBG level by 0.15 (SE = 0.07; *P* = 0.036). We also found that one unit increase in the natural log-transformed HbGA level was associated with an increase in the natural log-transformed serum AMH level by 0.31 (SE = 0.09; *P* = 0.003).

**Table 2:** Linear regression coefficients (S.E.) of sex hormones with a unit increase in ln-acrylamide and glycidamide adduct concentrations, with results weighted for sampling strategy.

	Unweighted no./ Population size	Ln HbAA (pmol/g Hb)	*P*	Unweighted no./ Population size	Ln HbGA (pmol/g Hb)	*P*
Ln AMH (ng/ml)						
Unadjusted	460/14985390	0.31(0.12)	0.017	447/14627589	0.32(0.09)	0.003
Adjusted	455/14798997	0.24(0.14)	0.118	442/14451196	0.31(0.09)	0.003
Ln Inhibin B (pg/ml)						
Unadjusted	452/14740305	0.14(0.05)	0.011	439/14392504	0.07(0.03)	0.041
Adjusted	447/14563912	0.10(0.05)	0.046	434/14216111	0.04(0.03)	0.104
Ln SHBG(nmol/L)						
Unadjusted	465/15088650	0.18(0.06)	0.007	451/14729272	-0.01(0.04)	0.855
Adjusted	460/14912257	0.15(0.07)	0.036	446/14552879	-0.05(0.04)	0.230
Ln total Testosterone (ng/mL)						
Unadjusted	465/15088650	0.24(0.06)	0.001	451/14729272	0.06(0.04)	0.134
Adjusted	460/14912257	0.11(0.06)	0.099	446/14552879	-0.01(0.04)	0.794
Ln free Testosterone (ng/mL)						
Unadjusted	465/15088650	0.13(0.07)	0.095	451/14729272	0.07(0.05)	0.179
Adjusted	460/14912257	0.00(0.08)	0.996	446/14552879	0.02(0.05)	0.782
Ln bioavailable Testosterone (ng/mL)						
Unadjusted	465/15088650	0.19(0.08)	0.028	451/14729272	0.11(0.059)	0.094
Adjusted	460/14912257	0.09(0.11)	0.441	446/14552879	0.06(0.075)	0.438
Ln Estradiol (pg/mL)						
Unadjusted	453/14994792	0.08(0.05)	0.097	439/14635413	0.02(0.04)	0.623
Adjusted	449/14824285	0.03(0.07)	0.703	435/14464906	-0.02(0.04)	0.659
Ln Androstanedione glucuronide (ng/mL)						
Unadjusted	464/15023095	-0.07(0.08)	0.398	468/14663717	-0.00(0.05)	0.951
Adjusted	459/14846702	-0.03(0.08)	0.665	445/14487324	0.03(0.03)	0.405

^a^, Model adjusted for age, race/ethnicity, BMI z score and smoking status

Abbreviations: AMH: anti-Mullerian hormone; SHBG: sex hormone binding globulin

Analyses restricted to non-smokers were performed in S3 Table. We found that participants aged between 12 to 19 years and those with lower caffeine intake were associated with higher AMH, while subjects aged between 12 to 19 years or self-recognized as Mexican Americans were associated with higher inhibin B concentrations. The basic demographics of the sample population and the association between the level of AMH and inhibin B are outlined in S1 and S2 Tables.

The linear regression coefficients of serum inhibin B and SHBG levels with a unit increase in the natural log-transformed HbAA levels in different sample subpopulations are shown in Tables 3 and 4. The β coefficient between HbAA and inhibin B was higher in those aged between 20–45 years old, non-Hispanic Caucasian individuals, non-active smokers, and BMI z-scores > 0.15. The β coefficient between HbAA and SHBG was higher in subjects aged ≧ 45 years, non-Hispanic Caucasians, active smokers, and BMI z-scores > 0.15. No significant correlation between HbAA and AMH (S4 Table). The linear regression coefficients of the serum AMH levels with a unit increase in the natural log-transformed HbGA levels in different subgroups are shown in Table 4. The β coefficient between HbGA and AMH was higher in the following subgroups: age between 20–45 years old, non-active smokers, and BMI z-scores > 0.15. The *P*-value for test for interaction between acrylamide adducts and age groups, acrylamide adducts and race, acrylamide adducts and smoking status, acrylamide adducts and BMI z score were all insignificant in all sex hormones. There was no association between acrylamide exposure and testosterone, estradiol, and androstanedione glucuronide (S5, S6 and S7 Tables).

**Table 3:** Linear regression coefficients (SE) between ln-HbAA, inhibin B, and SHBG in different subpopulations of sample subjects with results weighted for sampling strategy.

	Unweighted no./ Population size	Ln inhibin B (ng/ml)		Unweighted no./ Population size	Ln SHBG (ng/ml)	
		β coeff (S.E.)	P value		β coeff (S.E.)	P value
Age, y						
12–19	156/2088544	0.07 (0.01)	0.496	159/2145120	0.21 (0.12)	0.118
20–44	130/6679231	0.38 (0.12)	0.321	130/6735835	0.09 (0.09)	0.350
≧45	161/5796137	0.13 (0.07)	0.068	171/5796137	0.23 (0.11)	0.047
Race						
Non-Hispanic White	191/10201001	0.14 (0.05)	0.026	198/10490006	0.17 (0.07)	0.035
Others	256/4362911	0.02 (0.07)	0.757	262/4422251	0.09 (0.07)	0.215
Serum cotinine (ng/mL)						
<0.142	221/6473948	0.24 (0.08)	0.008	229/6650284	0.15 (0.13)	0.242
≧0.142	226/8089964	0.05 (0.07)	0.460	231/8261973	0.15 (0.06)	0.018
BMI z score						
≦ 0.15	225/8028661	0.08 (0.07)	0.267	230/8105091	0.08 (0.07)	0.270
>0.15	222/6535250	0.15 (0.09)	0.121	230/6807166	0.27 (0.07)	0.001

Model adjusted for age, race/ethnicity, BMI z score and smoking status

Abbreviations: BMI z score, z score of body mass index.

**Table 4:** Linear regression coefficients (SE) between ln-HbGA and AMH in different subpopulations of sample subjects with results weighted for sampling strategy.

	Unweighted no./ Population size	Ln anti-Mullerian hormone (ng/ml)	
		β coeff (S.E.)	P value
Age, y			
12–19	152/2033039	0.17 (0.08)	0.054
20–44	128/6696375	0.38 (0.12)	0.007
≧45	162/5721783	0.22 (0.13)	0.095
Race			
Non-Hispanic White	194/10248799	0.31 (0.10)	0.010
Others	248/4202397	0.31 (0.16)	0.066
Serum cotinine (ng/mL)			
<0.142	221/6469051	0.32 (0.20)	0.128
≧0.142	221/7982145	0.29 (0.10)	0.012
BMI z score			
≦ 0.15	225/7994319	0.23 (0.10)	0.040
>0.15	217/6456877	0.46 (0.18)	0.020

Model adjusted for age, race/ethnicity, BMI z score and smoking status

Abbreviations: AMH, anti-Mullerian hormone; BMI z score, z score of body mass index.

## Discussion

This report is the first epidemiologic study to investigate the association between acrylamide levels and sex hormone alternations in males in ages of adolescents and adults. In the present cross-sectional analysis of the general US population, we found that acrylamide exposure was associated with sex hormone alternations in male participants. The HbAA level was positively associated with the serum levels of inhibin B and SHBG, and the HbGA level was associated with an increase in the serum AMH level. There was no association between acrylamide exposure and other sex hormones, including testosterone, estradiol and androstanedione glucuronide.

Seminiferous tubules are composed of three major cell types: spermatogenic cells, Sertoli cells and Leydig cells. Sertoli cells, the epithelial supporting cells of developing spermatocytes in seminiferous tubules, produce several proteins including androgen binding protein, inhibin B and AMH. Additionally, Sertoli cells are responsible for DNA repair during spermatogenesis.[12] Leydig cells, the interstitial cells of seminiferous tubules, secret testosterone, androstenedione and dehydroepiandrosterone. Testis has been proposed as a target organ of acrylamide toxicity, specifically on Sertoli and Leydig cells.[13] During spermatogenesis, acrylamide might exert genotoxicity directly on DNA or indirectly by damaging Sertoli cell’s function of DNA repair. Alternatively, acrylamide may also affect reproduction by altering sex hormone homeostasis. Indeed, endocrine dysfunction has been observed in acrylamide-treated animals, especially in males.[14, 15]

Thus far, the associations between acrylamide exposure and reproductive toxicity in human is unclear. Epidemiological studies have observed positive associations between acrylamide intake and endometrial, ovarian and breast cancer,[1] which are all hormone-related. This suggests that acrylamide might affect hormone homeostasis, especially sex hormones. A nested case-control study using the Nurses’ Health Studies showed no associations between acrylamide intake and plasma levels of sex hormones or SHBG among premenopausal women overall.[6] In subgroup analysis, acrylamide intake was associated with luteal total and free estradiol levels among normal-weight pre-menopausal women. Among post-menopausal women, there was no association between acrylamide intake and any sex hormone, but a trend of lower estrone sulfate. Specifically, in overweight post-menopausal women, acrylamide intake was associated with higher testosterone and androstenedione.[6] In another Japanese study, acrylamide intake was inversely associated with total and free estradiol levels and positively associated with the FSH level in premenopausal women.[16] Another study found a significant positive association between acrylamide intake and urinary testosterone and androstenediol levels in boys aged 3–6 years.[17] This report suggested that acrylamide might alter androgens secreting from adrenal glands. However, we found acrylamide level was positively associated with serum level of inhibin B, SHBG, and AMH but not other sex hormones in the NHANES cohort. This discrepancy might be explained by the nature of the study design between studies including pre- or post-puberty and/or the samples (serum vs. urine) used for sex hormones measurement. Another explanation is that acrylamide exposure was measured by questionnaires in most epidemiologic studies. However, the correlations between questionnaire data and hemoglobin adduct levels only varied from absent or low to modest in previous studies. [18, 19] Measurement error in the acrylamide intake assessment will led to very high uncertainty. [6]

Inhibin B, a member of the transforming growth factor β (TGF-β) family, is secreted by Sertoli cells to promote spermatogenesis. It was proposed that serum inhibin B is positively correlated with function of Sertoli cells as well as spermatogenesis.[20] In our report, we observed a positive association between inhibin B levels and acrylamide exposure (HbAA). However, we did not observed association between acrylamide exposure and testosterone level. In men, testosterone produced by Leydig cells is under the control of LH, whereas FSH enhances the production of inhibin B by Sertoli cells. Inhibin B also controls FSH secretion via negative feedback mechanism. Although FSH level was not available in this current study, it is possible that the elevation of inhibin B after acrylamide intoxication would subsequently decrease FSH. [21] However, case reports on isolated FSH deficiency instead showed normal testosterone and LH levels. [21] Moreover, although it is not clear where in the hypothalamic-pituitary axis acrylamide may affect, acrylamide may also lead to FSH over-secretion from the pituitary gland. Previous epidemiologic study reported acrylamide intake has been positively associated with FSH levels in pre-menopausal women.[16] Thus, an increase in FSH levels may subsequently lead to inhibin B secretion. [22] the increase in FSH level may be a compensatory effect.

The observed positive association of acrylamide exposure with AMH levels is unexpected. Similar to inhibin B, AMH is a member of the TGF-β superfamily produced by Sertoli cells. Even though lower serum AMH level has been reported in sub-fertile men, it is inconclusive.[23, 24] Instead, the higher AMH in participants with higher HbGA does not necessarily suggest better fertility, and it might be a secondary response to sub-fertile status caused by acrylamide. Although an association between estradiol and acrylamide has been reported previously, the results were discordant between animal and human studies. The acrylamide intake was associated with decreased estradiol levels in female rats,[25] but with higher total and free luteal estradiol levels in pre-menopausal women.[6] Instead, we could not detect any association between acrylamide exposure and estradiol in our cohort. Although it is estimated that the level of acrylamide leading to reproductive toxicity in human would be much higher than the estimated dietary exposure, the cumulative effect of acrylamide on reproductive system cannot be excluded.[26]

SHBG, the principal transport protein for testosterone and estradiol, binds testosterone and dihydrotestosterone in men and estrogen in women. No animal study has investigated the association between acrylamide exposure, AMH and SHBG. The SHBG-bound sex hormones are inactive, thus higher SHBG levels suggests lesser active sex hormones. Thus, the positive association between HbAA and SHBG suggest a possible sub-functional sex hormones levels in acrylamide-exposure participants. Although no association between acrylamide exposure and various testosterone levels was found in the current study, we did observe a trend between HbAA and total testosterone (regression coefficient = 0.11 *P* = 0.099) but not free or bioavailable testosterone (regression coefficient = 0.00 *P* = 0.996 and regression coefficient = 0.09 *P* = 0.441, respectively). The trend between HbAA and total testosterone may be due to the contribution of SHBG. However, the non-significant result in total testosterone might be attributed to the relatively low population size of this cohort; even though this is a nation-wide representative cohort.

We previously have demonstrated that HbAA was negatively associated with BMI, fat mass,[27] reduced serum insulin and insulin resistance in the NHANES cohort.[2] It was reported that low serum SHBG concentrations were associated with insulin resistance and type 2 diabetes mellitus.[28, 29] Previous analyses have also identified that sex hormones were inversely correlated with adiposity (abdominal and visceral fat) in men.[30, 31] It is not known yet about the causal relationship between low concentrations of sex hormones and insulin resistance/fat mass.

In this study, we also identified that caffeine intakes are positively correlated with HbAA & HbGA. Acrylamide can be generated during the heating process of carbohydrate-containing food. Accordingly, it is plausible that the roasting process of coffee beans might lead to high acrylamide production. Subsequently, increased coffee consumption might lead to higher caffeine and possibly elevated acrylamide levels. Indeed, the correlation between coffee consumption and acrylamide level has been previously documented.[32] Mojska H *et al*. concludes that roasting process had the most significant effect on acrylamide levels in natural coffee.

Lastly, in this study, with limitations, we identified associations between acrylamide exposure and sex hormones, specifically in men. First, the cross-sectional design does not permit causal inference. Second, it is possible that acrylamide only serves as a surrogate for exposure to other chemicals. Third, we could not obtain information about amino acid intake, which affects the homeostasis and detoxification effect of glutathione.[33] Fourth, we use the age group between 12–19 to cover the puberty period in this current study. However, some participants are pre-pubertal, while others are adults. The individual difference will influence the sex hormone levels. Lastly, the information about fertility is lacking in this NHANES cohort.

In conclusion, we identified positive associations between acrylamide exposure and sex hormones (AMH, inhibin B, and SHBG) in a nationally representative survey of U.S. males. Since acrylamide exposure from food and smoking has become a worldwide concern, further longitudinal clinical and basic studies are urgently warranted to elucidate this putative causal relationship and its effect on reproductive toxicity in human.

## Supporting information

S1 Fig Flow chart of the study population (2003–2004 NHANES). (DOCX)

S1 Table The mean concentrations (95% C.I.) of sex hormones. (DOCX)

S2 Table The correlations between sex hormones. (DOCX)

S3 Table Basic demographics of the sample subjects including means (95% C.I.) of AMH and inhibin B concentrations. (DOCX)

S4 Table β coefficient (S.E.) of sex hormones with a unit increase in ln HbAA and HbGA concentrations in multiple linear regression analysis in non-smokers, with results weighted for sampling strategy. (DOCX)

S5 Table β coefficients (SE) between ln HbAA and AMH in different subpopulations of sample subjects in multiple linear analysis, with results weighted for sampling strategy. (DOCX)

S6 Table β coefficients (SE) between ln HbAA and estradiol in different subpopulations of sample subjects in multiple linear analysis, with results weighted for sampling strategy. (DOCX)

S7 Table β coefficients (SE) between ln HbAA and androstanedione glucuronide in different subpopulations of sample subjects in multiple linear analysis, with results weighted for sampling strategy. (DOCX)

S8 Table β coefficients (SE) between ln HbAA and total testosterone in different subpopulations of sample subjects in multiple linear analysis, with results weighted for sampling strategy. (DOCX)
