# Supplementary material for: Association between acrylamide exposure and sex hormones in males: NHANES, 2003–2004
Source: PLoS One. 2020 Jun 18;15(6):e0234622. doi: 10.1371/journal.pone.0234622 (PMC7302712; doi:10.1371/journal.pone.0234622)
Supplement: S1 Fig — (DOCX) [file pone.0234622.s001.docx]

**Supplementary Figure 1. Flow chart of the study population (2003-2004 NHANES)**

Enrolled NHANES 2003-2004 population (n=10,122)

n=3,021

**No**

Availability HbAA or HbGA

Availability testosterone or SHBG or estradiol or androstenedione glucuronide or AMH or inhibin B for men

n=6,633

**No**

**Yes**

**Yes**

Final analysis population (n=468)

**No**
