# Supplementary material for: Association between acrylamide exposure and sex hormones in males: NHANES, 2003–2004
Source: PLoS One. 2020 Jun 18;15(6):e0234622. doi: 10.1371/journal.pone.0234622 (PMC7302712; doi:10.1371/journal.pone.0234622)
Supplement: S1 Table — (DOCX) [file pone.0234622.s002.docx]

**Supplementary table 1. The mean concentrations (95% C.I.) of sex hormones**

|  | n | Mean | 95% C.I. |
| --- | --- | --- | --- |
| AMH (ng/ml) | 460 | 10.46 | 8.77-12.14 |
| Inhibin B (pg/ml) | 452 | 140.50 | 134.71-146.28 |
| SHBG(nmol/L) | 465 | 38.99 | 36.68-41.30 |
| Total Testosterone (ng/mL) | 465 | 5.23 | 4.93-5.52 |
| Estradiol (pg/mL) | 465 | 39.50 | 36.81-42.19 |
| Androstanedione glucuronide (ng/mL) | 464 | 7.49 | 7.04-7.93 |
