# Supplementary material for: Association between acrylamide exposure and sex hormones in males: NHANES, 2003–2004
Source: PLoS One. 2020 Jun 18;15(6):e0234622. doi: 10.1371/journal.pone.0234622 (PMC7302712; doi:10.1371/journal.pone.0234622)
Supplement: S2 Table — (DOCX) [file pone.0234622.s003.docx]

**Supplementary table 2. The correlations between sex hormones**

|  |  | AMH (ng/ml) | Inhibin B (pg/ml) | SHBG  (nmol/L) | Total Testosterone (ng/mL) | Estradiol (pg/mL) | Androstanedione glucuronide (ng/mL) |
| --- | --- | --- | --- | --- | --- | --- | --- |
| AMH (ng/ml) | β coefficients | 1 | 0.409 | 0.038 | -0.014 | -0.152 | -0.153 |
|  | *P* value |  | <0.001 | 0.423 | 0.763 | 0.001 | 0.001 |
| Inhibin B (pg/ml) | β coefficients |  | 1 | 0.050 | 0.148 | -0.107 | -0.038 |
|  | *P* value |  |  | 0.288 | 0.002 | 0.023 | 0.425 |
| SHBG(nmol/L) | β coefficients |  |  | 1 | 0.163 | -0.200 | -0.202 |
|  | *P* value |  |  |  | <0.001 | <0.001 | <0.001 |
| Total Testosterone (ng/mL) | β coefficients |  |  |  | 1 | 0.523 | 0.284 |
|  | *P* value |  |  |  |  | <0.001 | <0.001 |
| Estradiol (pg/mL) | β coefficients |  |  |  |  | 1 | 0.376 |
|  | *P* value |  |  |  |  |  | <0.001 |
| Androstanedione glucuronide (ng/mL) | β coefficients |  |  |  |  |  | 1 |
|  | *P* value |  |  |  |  |  |  |

Model adjusted for age, race/ethnicity, smoking status, BMI z score
