# Supplementary material for: Association between acrylamide exposure and sex hormones in males: NHANES, 2003–2004
Source: PLoS One. 2020 Jun 18;15(6):e0234622. doi: 10.1371/journal.pone.0234622 (PMC7302712; doi:10.1371/journal.pone.0234622)
Supplement: S3 Table — (DOCX) [file pone.0234622.s004.docx]

**Supplement table 3. Basic demographics of the sample subjects including means (95% C.I.) of AMH and inhibin B concentrations**

|  | Unweighted no. (%) | AMH  (ng/mL) | *P* value between groups | Unweighted no. (%) | Inhibin B  (ng/mL) | *P* value between groups |
| --- | --- | --- | --- | --- | --- | --- |
| Overall | 460(100) | 10.46(8.77-12.14) |  | 452(100) | 140.50 (134.71-146.28) |  |
| Age, y |  |  | <0.001 |  |  | <0.001 |
| 12-19 | 159 (34.5) | 15.31 (10.82-19.80) |  | 158 (35.0) | 162.95 (152.75-173.16) |  |
| 20-59 | 197 (42.8) | 9.34 (8.04-10.65) |  | 197 (43.6) | 137.44 (129.09-145.79) |  |
| ≧60 | 104 (22.7) | 5.16 (4.43-5.89) |  | 97 (21.4) | 110.12 (100.05-120.20) |  |
| Race |  |  | 0.085 |  |  | 0.024 |
| Mexican American | 120 (26.1) | 13.69 (8.81-18.57) |  | 119 (26.3) | 154.07 (143.03-165.12) |  |
| Non-Hispanic White | 198 (43.0) | 8.01 (5.94-10.10) |  | 192 (42.5) | 135.75 (127.01-144.49) |  |
| Non-Hispanic Black | 110 (23.9) | 11.78 (9.25-14.31) |  | 110 (24.4) | 132.10 (120.26-143.95) |  |
| Others Hispanic | 16 (3.5) | 7.61(4.78-10.46) |  | 16 (3.5) | 163.10 (119.37-206.84) |  |
| Other race | 16 (3.5) | 10.14 (3.98-16.31) |  | 15(3.3) | 130.98 (103.01-158.95) |  |
| BMI z score |  |  | 0.607 |  |  | 0.099 |
| ≦ 0.15 | 231 (50.8) | 10.92 (8.08-13.75) |  | 225 (50.3) | 145.43 (136.92-153.95) |  |
| >0.15 | 224 (49.2) | 10.02 (8.17-11.88) |  | 222 (49.7) | 135.63(127.64-143.63) |  |
| Smoking |  |  | 0.073 |  |  | 0.107 |
| Nonexposed | 64 (13.9) | 15.10 (6.56-23.65) |  | 60 (13.2) | 130.85 (115.67-146.03) |  |
| Expose to ETS | 258 (56.1) | 9.24 (7.77-10.71) |  | 255 (56.6) | 138.08 (130.72-145.44) |  |
| Active smoker | 138 (30.0) | 10.58 (7.62-13.55) |  | 137 (30.2) | 149.21 (137.63-160.79) |  |
| Caffeine intake(mg/day) |  |  | 0.006 |  |  | 0.625 |
| < 72 | 202 (49.9) | 12.43 (9.25-15.60) |  | 200 (50.3) | 140.24(131.96-148.88) |  |
| ≧72 | 203 (50.1) | 7.71 (6.65-8.77) |  | 198 (49.7) | 137.37(128.46-146.29) |  |
