# Supplementary material for: Association between acrylamide exposure and sex hormones in males: NHANES, 2003–2004
Source: PLoS One. 2020 Jun 18;15(6):e0234622. doi: 10.1371/journal.pone.0234622 (PMC7302712; doi:10.1371/journal.pone.0234622)
Supplement: S4 Table — (DOCX) [file pone.0234622.s005.docx]

**Supplementary table 4. β coefficient (S.E.) of sex hormones with a unit increase in ln HbAA and HbGA concentrations in multiple linear regression analysis in non-smokers, with results weighted for sampling strategy**

|  | Unweighted no./ Population size | Ln HbAA  (pmol/g Hb) | *P* | Unweighted no./ Population size | Ln HbGA  (pmol/g Hb) | *P* |
| --- | --- | --- | --- | --- | --- | --- |
| Ln AMH (ng/ml) |  |  |  |  |  |  |
| Unadjusted | 322/9174565 | 0.40(0.27) | 0.153 | 312/8918360 | 0.34(0.15) | 0.038 |
| Adjusted | 318/9022206 | 0.44(0.30) | 0.166 | 308/8766001 | 0.38(0.15) | 0.022 |
| Ln inhibin B (pg/ml) |  |  |  |  |  |  |
| Unadjusted | 315/8955000 | 0.22(0.05) | 0.001 | 305/8690795 | 0.04(0.06) | 0.526 |
| Adjusted | 311/8802641 | 0.16(0.06) | 0.020 | 301/8546436 | 0.04(0.03) | 0.303 |
| Ln SHBG(nmol/L) |  |  |  |  |  |  |
| Unadjusted | 325/9173411 | 0.16(0.12) | 0.220 | 314/8905628 | -0.08(0.06) | 0.187 |
| Adjusted | 321/9021053 | 0.11(0.13) | 0.410 | 310/8753270 | -0.08(0.05) | 0.087 |
| Ln total Testosterone (ng/mL) |  |  |  |  |  |  |
| Unadjusted | 325/9173411 | 0.14(0.14) | 0.347 | 314/8905628 | -0.06(0.05) | 0.274 |
| Adjusted | 321/9021053 | 0.05(0.12) | 0.696 | 310/8753270 | -0.06(0.05) | 0.292 |
| Ln free Testosterone (ng/mL) |  |  |  |  |  |  |
| Unadjusted | 325/9173411 | 0.02(0.18) | 0.926 | 314/8905628 | -0.01(0.08) | 0.897 |
| Adjusted | 321/9021053 | -0.03(0.16) | 0.856 | 310/8753270 | 0.00(0.07) | 0.979 |
| Ln bioavailable Testosterone (ng/mL) |  |  |  |  |  |  |
| Unadjusted | 325/9173411 | 0.23(0.23) | 0.336 | 314/8905628 | 0.05(0.11) | 0.675 |
| Adjusted | 321/9021053 | 0.17(0.23) | 0.478 | 310/8753270 | 0.05(0.10) | 0.607 |
| Ln estradiol (pg/mL) |  |  |  |  |  |  |
| Unadjusted | 313/9079553 | -0.01(0.12) | 0.946 | 302/8811770 | -0.04(0.05) | 0.428 |
| Adjusted | 310/8933080 | -0.01(0.12) | 0.913 | 299/8665297 | -0.04(0.06) | 0.531 |
| Ln androstanedione glucuronide (ng/mL) |  |  |  |  |  |  |
| Unadjusted | 324/9107856 | -0.09(0.11) | 0.431 | 313/8840073 | 0.04(0.04) | 0.336 |
| Adjusted | 320/8955498 | -0.10(0.10) | 0.344 | 309/8687715 | 0.03(0.03) | 0.432 |

Model adjusted for age, race/ethnicity, smoking status, BMI z score

Abbreviations: AMH: anti-Mullerian hormone; HbAA, hemoglobin adducts of acrylamide; HbGA, hemoglobin adducts of glycidamide; SHBG: sex hormone binding globulin; Ln, natural logarithm; S.E., standard error.
