# Supplementary material for: Association between acrylamide exposure and sex hormones in males: NHANES, 2003–2004
Source: PLoS One. 2020 Jun 18;15(6):e0234622. doi: 10.1371/journal.pone.0234622 (PMC7302712; doi:10.1371/journal.pone.0234622)
Supplement: S5 Table — (DOCX) [file pone.0234622.s006.docx]

**Supplementary table 5. β coefficients (SE) between ln HbAA and AMH in different subpopulations of sample subjects in multiple linear analysis, with results weighted for sampling strategy**

|  | Unweighted no./ Population size | Ln AMH (ng/ml) | |
| --- | --- | --- | --- |
|  |  | β coefficient (S.E.) | *P* value |
| Age, y |  |  |  |
| 12-19 | 157/2095247 | 0.15 (0.15) | 0.327 |
| 20-44 | 131/6780430 | 0.28 (0.17) | 0.125 |
| ≧45 | 167/5923320 | 0.05 (0.18) | 0.791 |
| Race |  |  |  |
| Non-Hispanic White | 197/10404195 | 0.25 (0.16) | 0.157 |
| Others | 258/4394802 | 0.21 (0.24) | 0.387 |
| Serum cotinine (ng/mL) |  |  |  |
| <0.142 | 318/9022206 | 0.45 (0.31) | 0.169 |
| ≧0.142 | 137/5776790 | 0.13 (0.16) | 0.438 |
| BMI z score |  |  |  |
| ≦ 0.15 | 231/8168757 | 0.28 (0.17) | 0.120 |
| >0.15 | 224/6630240 | 0.21 (0.22) | 0.344 |

Model adjusted for age, race/ethnicity, BMI z score and smoking status

Abbreviations: AMH: anti-Mullerian hormone; BMI z score, z score of body mass index; HbAA, hemoglobin adducts of acrylamide; Ln, natural logarithm; S.E., standard error.
