# Supplementary material for: Association between acrylamide exposure and sex hormones in males: NHANES, 2003–2004
Source: PLoS One. 2020 Jun 18;15(6):e0234622. doi: 10.1371/journal.pone.0234622 (PMC7302712; doi:10.1371/journal.pone.0234622)
Supplement: S6 Table — (DOCX) [file pone.0234622.s007.docx]

**Supplementary table 6. β coefficients (SE) between ln HbAA and estradiol in different subpopulations of sample subjects in multiple linear analysis, with results weighted for sampling strategy**

|  | Unweighted no./ Population size | Ln AMH (ng/ml) | |
| --- | --- | --- | --- |
|  |  | β coefficient (S.E.) | *P* value |
| Age, y |  |  |  |
| 12-19 | 148/2057147 | -0.07 (0.16) | 0.687 |
| 20-44 | 130/6735835 | 0.03 (0.10) | 0.755 |
| ≧45 | 171/6031303 | 0.03 (0.08) | 0.747 |
| Race |  |  |  |
| Non-Hispanic White | 197/10456161 | 0.00 (0.08) | 0.973 |
| Others | 252/4368124 | 0.10 (0.07) | 0.200 |
| Serum cotinine (ng/mL) |  |  |  |
| <0.142 | 310/8933080 | -0.02 (0.11) | 0.839 |
| ≧0.142 | 139/5891205 | 0.07 (0.05) | 0.210 |
| BMI z score |  |  |  |
| ≦ 0.15 | 225/8050776 | 0.05 (0.08) | 0.506 |
| > 0.15 | 224/6773509 | -0.01 (0.10) | 0.951 |

Model adjusted for age, race/ethnicity, BMI z score and smoking status

Abbreviations: BMI z score, z score of body mass index; HbAA, hemoglobin adducts of acrylamide; Ln, natural logarithm; S.E., standard error.
