# Supplementary material for: Association between acrylamide exposure and sex hormones in males: NHANES, 2003–2004
Source: PLoS One. 2020 Jun 18;15(6):e0234622. doi: 10.1371/journal.pone.0234622 (PMC7302712; doi:10.1371/journal.pone.0234622)
Supplement: S7 Table — (DOCX) [file pone.0234622.s008.docx]

**Supplementary table 7. β coefficients (SE) between ln HbAA and androstanedione glucuronide in different subpopulations of sample subjects in multiple linear analysis, with results weighted for sampling strategy**

|  | Unweighted no./ Population size | Ln AMH (ng/ml) | |
| --- | --- | --- | --- |
|  |  | β coefficient (S.E.) | *P* value |
| Age, y |  |  |  |
| 12-19 | 159/2145120 | -0.14 (0.13) | 0.326 |
| 20-44 | 130/6735835 | -0.02 (0.09) | 0.831 |
| ≧45 | 170/5965748 | -0.02 (0.13) | 0.864 |
| Race |  |  |  |
| Non-Hispanic White | 197/10424451 | -0.02 (0.08) | 0.849 |
| Others | 262/4422251 | -0.07 (0.09) | 0.471 |
| Serum cotinine (ng/mL) |  |  |  |
| <0.142 | 320/8955498 | -0.09 (0.10) | 0.384 |
| ≧0.142 | 139/5891205 | 0.00 (0.10) | 0.989 |
| BMI z score |  |  |  |
| ≦ 0.15 | 230/8105091 | -0.03 (0.08) | 0.702 |
| > 0.15 | 229/6741611 | -0.05 (0.11) | 0.638 |

Model adjusted for age, race/ethnicity, BMI z score and smoking status

Abbreviations: BMI z score, z score of body mass index; HbAA, hemoglobin adducts of acrylamide; Ln, natural logarithm; S.E., standard error.
