# Supplementary material for: Association between acrylamide exposure and sex hormones in males: NHANES, 2003–2004
Source: PLoS One. 2020 Jun 18;15(6):e0234622. doi: 10.1371/journal.pone.0234622 (PMC7302712; doi:10.1371/journal.pone.0234622)
Supplement: S8 Table — (DOCX) [file pone.0234622.s009.docx]

**Supplementary table 8. β coefficients (SE) between ln HbAA and total testosterone in different subpopulations of sample subjects in multiple linear analysis, with results weighted for sampling strategy**

|  | Unweighted no./ Population size | Ln total testosterone (ng/ml) | |
| --- | --- | --- | --- |
|  |  | β coefficient (S.E.) | *P* value |
| Age, y |  |  |  |
| 12-19 | 159/2145120 | -0.07 (0.29) | 0.819 |
| 20-44 | 130/6735835 | 0.04 (0.08) | 0.646 |
| ≧45 | 171/5721783 | 0.20 (0.06) | 0.008 |
| Race |  |  |  |
| Non-Hispanic White | 198/10490006 | 0.12 (0.08) | 0.152 |
| Others | 262/4422251 | 0.07 (0.04) | 0.074 |
| Serum cotinine (ng/mL) |  |  |  |
| <0.142 | 321/9021053 | 0.05 (0.12) | 0.692 |
| ≧0.142 | 139/5891205 | 0.15 (0.04) | 0.001 |
| BMI z score |  |  |  |
| ≦ 0.15 | 230/8105091 | 0.11 (0.03) | 0.005 |
| >0.15 | 230/6807166 | 0.13 (0.14) | 0.340 |

Model adjusted for age, race/ethnicity, BMI z score and smoking status

Abbreviations: BMI z score, z score of body mass index; HbAA, hemoglobin adducts of acrylamide; Ln, natural logarithm; S.E., standard error.
